# Primary Myeloid Sarcoma of the Prostate: A Case Report and Literature Review

**DOI:** 10.1155/2018/3604298

**Published:** 2018-04-29

**Authors:** Ryan Nguyen, Hamid Sayar

**Affiliations:** ^1^Department of Medicine, Indiana University School of Medicine, Indianapolis, IN, USA; ^2^Division of Hematology and Oncology, Department of Medicine, Indiana University School of Medicine, Indianapolis, IN, USA

## Abstract

We report the case of a 73-year-old male with primary myeloid sarcoma (MS) of the prostate. He underwent remission-induction chemotherapy followed by conventional consolidation for acute myeloid leukemia (AML). One year after initial diagnosis, he was without evidence of AML, the longest reported period of time in the literature for a case of primary MS of the prostate. From 1985 to 2017, fifteen other cases of MS of the prostate have been reported and are reviewed here. Five cases occurred as primary MS, without evidence of AML on bone marrow examination or prior history of hematologic disorders, and progressed to AML within a range of three weeks to seven months. None of these cases were started on conventional chemotherapy for AML prior to progression. Due to its rarity, primary MS of the prostate is often diagnosed incidentally, but prompt AML-targeted treatment is crucial to delaying the progression to AML.

## 1. Introduction

Myeloid sarcoma (MS) is an uncommon extramedullary tumor of immature myeloid cells, occurring in 2.4–9.1% of patients with AML [1–3]. First described as “chloroma” in 1853 due to its green color associated with myeloperoxidase (MPO), the phenomenon was later known as “granulocytic sarcoma” to describe tumors of granulocytic origin [4]. More recently, MS has been used to describe any tumor related to acute leukemia or myelodysplastic syndromes (MDSs). MS typically occurs concurrently with or following the onset of systemic bone marrow leukemia, known as secondary MS. Uncommonly, MS can precede bone marrow involvement [5]. Primary MS is defined by MS in the absence of a history of leukemia, MDS, or myeloproliferative neoplasm and negative bone marrow examination. Median time to development of AML from presentation of primary MS ranges from five to twelve months [6].

The most common sites of MS involvement are the soft tissue, bone, periosteum, and lymph nodes [6]. MS of the prostate is extremely uncommon and described in only limited case reports. A 2016 literature review by Koppisetty et al. found eight cases of MS of the prostate from 1997 to 2014, four of which were primary MS. Time to development of AML in primary MS of the prostate ranged from three weeks to four months [7]. We present a case of primary MS of the prostate placed on standard remission-induction chemotherapy and without evidence of AML one year later.

## 2. Case Presentation

A 73-year-old man presented to a urology clinic with urinary frequency and hesitancy. His past medical history was significant for diabetes mellitus type II, benign prostatic hypertrophy, and chronic kidney disease stage II. He had no prior known history of malignancy. His physical exam showed no lymphadenopathy or palpable enlargement of the liver or spleen. A complete blood count revealed a hemoglobin count of 13.3 gm/dl, a white blood cell count of 5.8 × 10^9^/l, and a platelet count of 245 × 10^9^/l. He was initially suspected to have benign prostatic hypertrophy and underwent transurethral resection of the prostate (TURP). However, four out of fifty-three prostate chips obtained during TURP were found to have diffuse proliferation of large atypical cells positive for MPO, CD34, and CD68, with Ki-67 of 100%. He was diagnosed with MS of the prostate based on morphological findings.

**Figure 1:**
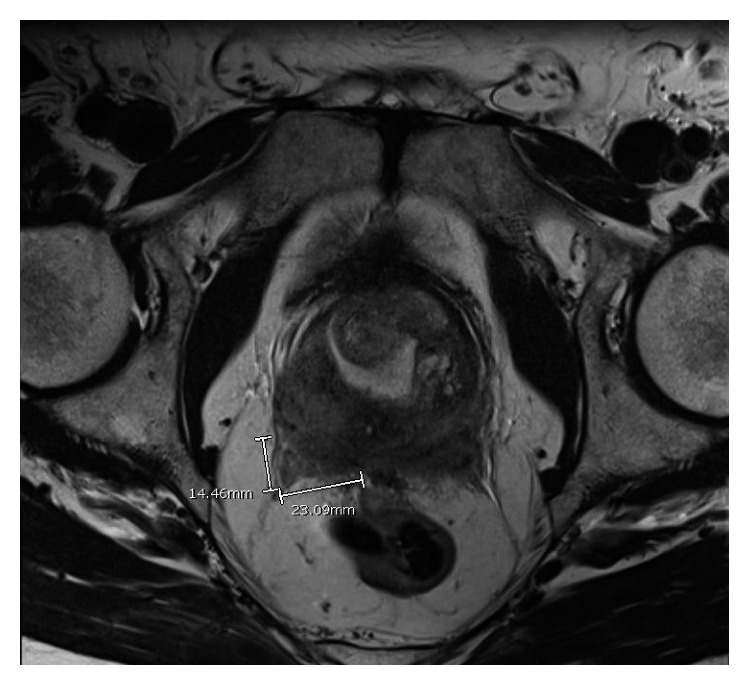
Pelvic MRI at diagnosis (axial view).

Positron emission tomography-computed tomography (PET-CT) was performed to screen for additional sites of involvement but did not show any suspicious lesions. Magnetic resonance imaging (MRI) of the pelvis with contrast, performed two weeks after PET-CT, demonstrated a 2.3 cm lesion in the central zone at the base of the prostate with concern for the surrounding tissue invasion (Figure 1). Bone marrow examination including aspiration and biopsy which included flow cytometry performed soon afterward did not show any evidence of AML or abnormal myeloblasts.

**Figure 2:**
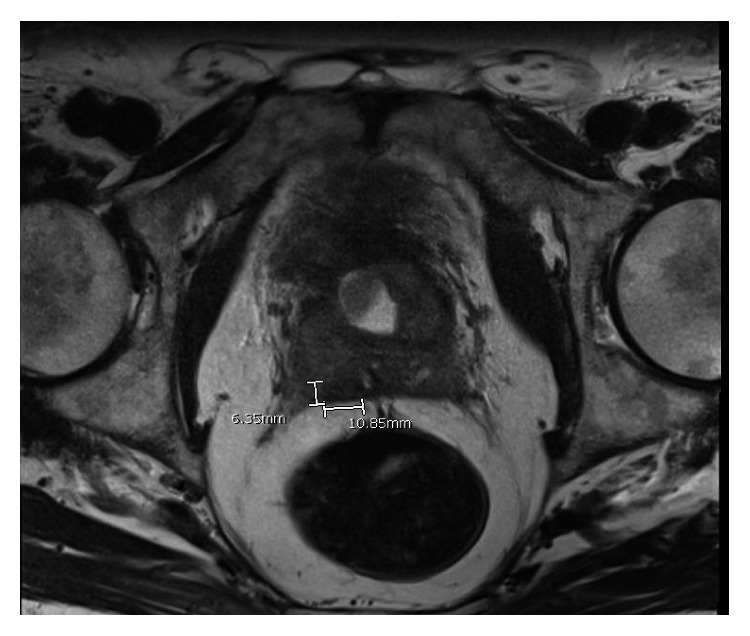
Pelvic MRI after treatment (axial view).

The patient was started on AML remission-induction chemotherapy with “7 + 3” regimen, consisting of cytarabine and idarubicin. Postinduction pelvis MRI showed decrease in the size of the prostate mass to 1.5 cm. He subsequently received three cycles of consolidation chemotherapy with intermediate-dose cytarabine; serial imaging during treatments showed continuous response without complete resolution of the prostate lesion (Figure 2). Maximum response was achieved with the second consolidation therapy. He is now on active surveillance with serial pelvis MRIs and labs every three months, without evidence of systemic relapse or pelvic progression at one year from original diagnosis.

## 3. Methods

A comprehensive literature review was performed through the PubMed database from 1985 to 2017 using the keywords myeloid sarcoma, granulocytic sarcoma, chloroma, extramedullary leukemic infiltration, and prostate. Articles were screened for the abovementioned terms, specific for myeloid lineage tumors including AML, MDS, and myeloproliferative disorders/neoplasms.

## 4. Results

A total of fifteen cases of myeloid sarcoma of the prostate were found in the literature search as outlined in Tables 1 and 2. Five were diagnosed as primary MS and ten as secondary MS. The age of presentation at diagnosis of MS was between 8 and 79 years, with a median age of 66.5 years.

Of the primary MS cases, none were started on chemotherapy for AML at the time of diagnosis due to either initial misdiagnosis or receiving local RT as initial treatment instead. Case 1 died shortly after undergoing TURP and prior to systemic treatment. Cases 2 and 3 were misdiagnosed as lymphoma and sarcoma, respectively, and progressed to AML within seven months. Case 2 achieved CR and was alive 19 months after diagnosis, and case 3 died three months after diagnosis. Both cases 4 and 5 were treated with local RT initially and progressed to AML within four months. After starting chemotherapy upon AML progression, both cases 4 and 5 achieved CR. Time to development of systemic disease ranged from three weeks to seven months. Our case represents the longest reported period from the diagnosis of primary MS of the prostate without development of systemic disease at one year. Ours is also the first case started on chemotherapy for AML at the time of discovery of primary MS of the prostate.

Secondary MS cases were split between six (cases 7–12) with concurrent AML and four (cases 13–16) with a previous history of AML or a clonal hematologic disorder. Patients with concurrent systemic disease were all started on chemotherapy upon diagnosis. Three (cases 7–9) died within 6 months of diagnosis, one died shortly after initiating chemotherapy (case 10), one (case 11) had no follow-up information, and one (case 12) achieved a CR. Treatment options differed among secondary MSs with the previous history of systemic disease, with time from the original disease to discovery of secondary MS ranging from six months to nine years. Two cases (cases 13-14) underwent only RT to the prostate bed at the time of MS of the prostate discovery. No follow-up information was available on case 13, but case 14 experienced AML relapse just two months after secondary MS discovery. He was started on systemic chemotherapy and was alive five months later. Case 15 underwent both systemic chemotherapy and RT at the time of discovery of MS but developed AML relapse with fatal outcome six months later. Case 16 was not started on systemic therapy at the time of MS diagnosis and developed AML relapse four months later, at which point he was treated with systemic chemotherapy and achieved CR. The two cases (cases 13 and 16) who did not receive systemic chemotherapy at the time of discovery of secondary MS had AML relapse within four months.

## 5. Discussion

MS is a very uncommon manifestation of AML, reported in 2.5–9.1% of these patients [1–3]. Patients with MS most often have clinical evidence of concurrent AML involving the bone marrow and/or blood. Primary MS with no evidence of AML is rare [5]. The underlying molecular basis of MS is thought to be aberrant chemokine or adhesion molecular expression in leukemic blasts [21]. A total of 15 cases of MS involving the prostate gland have been reported in the literature, of which 6 (40%) presented without prior or concurrent evidence of AML. Fourteen out of 15 (93.3%) cases originally presented with lower urinary tract symptoms that warranted further work-up, often including TURP, leading to tissue diagnosis.

Diagnosis of MS of the prostate poses a challenging task due to the rarity of the condition. Definitive diagnosis requires a prostate tissue pathology evaluation with identification of myeloid precursor cells. Of the sixteen cases reviewed above, a tissue diagnosis was obtained via TURP for nine (56.2%), prostate biopsy for five (31.2%), and prostatectomy for two (12.5%). When possible, immunohistochemistry, flow cytometry, and cytogenetics studies can aid in the diagnosis. The most common immunohistochemical markers in MS are MPO, CD68, and lysozyme [6]. Once the diagnosis of MS is made, a bone marrow examination should be obtained to evaluate for AML. MS typically presents as a soft tissue mass that is best suited to imaging by PET-CT which can also be used for monitoring response to treatment [6]. Interestingly, in our case, the PET-CT scan did not reveal the prostatic lesion. However, MRI of the pelvis did show the suspected prostate mass which reduced in size with serial MRIs following initiation of treatment.

Current recommended treatment for MS with or without evidence of systemic disease is standard therapy for AML (remission-induction chemotherapy) [6]. Surgical resection or RT may be considered for local control but alone will not lead to long-term remission of the disease. Delayed or inadequate treatment of primary MS almost always results in more rapid progression to AML [22]. In a review of different treatment outcomes for 72 cases of primary MS, median time to development of AML is 12 months for those receiving chemotherapy for AML versus six months for only local radiation and three months for biopsy or surgical resection [23]. Our review of reported cases found the time to development of AML in primary MS of the prostate ranging from three weeks to seven months. None of these reported cases were started on standard therapy for AML until they had progressed to systemic disease. Our case, at one year, represents the longest progression-free period and the only reported case of primary MS of the prostate started on therapy for AML at the time of initial diagnosis of MS of the prostate.

Data on the prognosis of MS are limited, with estimated 5-year survival rates between 20% and 30%, similar to AML [24]. A 2006 study compared the outcomes of 16 patients with primary MS with those of a larger cohort of AML who underwent standard treatment [25]. When matched for cytogenetics and age, isolated MS was associated with improved event-free survival and overall survival on standard AML therapy. The better outcomes, however, may be multifactorial including the fact that these patients usually do not die until development of AML, or that they experience less treatment-related morbidity and mortality due to normal marrow hematopoiesis at the initiation of therapy.

MS of the prostate is rare, with 15 reported cases in the literature from 1985 to 2017 and only 5 cases of primary MS of the prostate. Patients typically present with lower urinary tract symptoms, and the diagnosis is often made incidentally. Definitive diagnosis is made by tissue diagnosis, and a bone marrow examination is needed afterwards to evaluate for AML. MRI of the pelvis can be useful for initial evaluation and monitoring response to treatment if PET-CT is nonrevealing. Primary MS of the prostate, similar to other cases of MS, should be treated with AML-type systemic chemotherapy promptly. Early initiation of appropriate therapy is key in delaying progression to AML and prolonging survival.

## Figures and Tables

**Table 1:** Primary myeloid sarcoma of the prostate.

Case	Age	Symptoms	Hematological diagnosis	Time to progression to AML	Treatment	Outcomes
1 [8]	79	Urinary retention	N/A	N/A	None	Died shortly after TURP
2 [9]	67	Urinary retention	AML-M2 (FAB classification)	7 months	Lymphoma-directed chemotherapy and then remission-induction chemotherapy	Originally misdiagnosed as lymphoma and received CHOP. Started remission-induction chemotherapy at AML relapse and achieved CR. Alive at 19 months after MS diagnosis
3 [10]	32	Dysuria, weight loss, and night sweats	AML-M5 (FAB classification)	3 weeks	Remission-induction chemotherapy	Died 3 months after MS diagnosis
4 [11]	71	Dysuria	AML	4 months	Chemotherapy (unspecified)	CR and further follow-up N/A
5 [12]	71	Dysuria	AML-M2 (FAB classification)	4 months	Remission-induction chemotherapy and then salvage chemotherapy	CR and then relapse with salvage chemotherapy with second CR; died 24 months after MS diagnosis due to brain hemorrhage
6 (present case)	73	Frequency and hesitancy	None	N/A	Remission-induction chemotherapy followed by consolidation	Without progression to AML at 1 year after MS diagnosis

AML: acute myeloid leukemia; CHOP: cyclophosphamide; doxorubicin, vincristine, and prednisone; CR: complete remission; FAB classification: French-American-British classification; MS: myeloid sarcoma; N/A: not available; TURP: transurethral resection of the prostate.

**Table 2:** Secondary myeloid sarcoma of the prostate.

Case	Age	Symptoms	Hematological diagnosis	Time from systemic disease to MS	Treatment	Outcomes
7 [13]	61	Hematuria and frequency	AML	Diagnosed simultaneously	Doxorubicin, vincristine, cytarabine, and prednisone	Died 5.5 months after MS diagnosis
8 [14]	65	Urinary retention and weight loss	AML-M4 (FAB classification)	Diagnosed simultaneously	Chemotherapy (unspecified)	Died 3 weeks after MS diagnosis
9 [15]	72	Urinary retention	CMML	Diagnosed simultaneously	Etoposide	CMML progressed to AML at 3 months and then died at 4 months after MS diagnosis
10 [16]	N/A	Urinary retention	AML	Diagnosed simultaneously	Chemotherapy (unspecified)	Died; no details
11 [17]	8	Urinary retention	AML-M2 (FAB classification)	Diagnosed simultaneously	High-dose cytarabine	N/A
12 [18]	44	Lower urinary tract symptoms	AML	Diagnosed simultaneously	Chemotherapy and SCT	CR and further follow-up N/A
13 [13]	72	Urinary retention and testicular pain	MDS	11 months	RT to the prostate bed for hematuria	N/A
14 [19]	65	Asymptomatic, indurated PSA	AML-M5 (FAB classification)	13 months	RT and then salvage chemotherapy	Initially treated with RT, developed AML relapse at 2 months, and underwent salvage chemotherapy. Alive at 5 months after MS diagnosis
15 [20]	68	Urinary retention	AML-M2 (FAB classification)	9 years	RT and chemotherapy (unspecified)	Progressed to AML at 6 months after MS diagnosis and died during salvage chemotherapy shortly after
16 [6]	66	Asymptomatic, rising PSA	MDS status after SCT	6 months	Induction chemotherapy and donor lymphocyte infusion	CR and alive at 1 year after MS diagnosis

AML: acute myeloid leukemia; CMML: chronic myelomonocytic leukemia; FAB classification: French-American-British classification; MDS: myelodysplastic syndrome; MS: myeloid sarcoma; N/A: not available; PSA: prostate-specific antigen; SCT: stem cell transplant.
